# Do triple test results predict risk for neonatal hyperbilirubinemia?

**DOI:** 10.12669/pjms.334.12420

**Published:** 2017

**Authors:** Hale Goksever Celik, Engin Celik, Gokhan Yildirim, Merih Cetinkaya

**Affiliations:** 1 Dr. Hale Goksever Celik, MD. Department of Obstetrics and Gynecology, Kanuni Sultan Suleyman Training and Research Hospital, Istanbul, Turkey; 2 Dr. Engin Celik MD. Department of Obstetrics and Gynecology, Istanbul University School of Medicine, Istanbul, Turkey; 3 Gokhan Yildirim, MD. Assistant Professor, Department of Obstetrics and Gynecology, Kanuni Sultan Suleyman Training and Research Hospital, Istanbul, Turkey; 4 Merih Cetinkaya, Assistant Professor, Department of Neonatology, Kanuni Sultan Suleyman Training and Research Hospital, Istanbul, Turkey

**Keywords:** Maternal screening, Neonatal hyperbilirubinemia, Triple test, Screening tests, Estriol

## Abstract

**Objective:**

Neonatal jaundice is the most common condition that requires hospital admission and outpatient follow-up after discharge in neonates. The values of more than 17 mg/dL in term infants are accepted as neonatal significant hyperbilirubinemia. We aimed to define if there is any relationship between second trimester serum markers and neonatal severe hyperbilirubinemia to protect the neonates from its neurological damage.

**Methods:**

Total 1372 pregnant women were enrolled who had done triple test between April 2014 and 2015 and then given birth at our hospital. Our primary outcome was neonatal significant hyperbilirubinemia.

**Results:**

The mean age of our study population was 27.9±5.6. A total of 59 patients had babies with neonatal hyperbilirubinemia after exclusion of Rh incompatibility. We detected that the presence of in vitro pregnancy, maternal health problems or poor obstetric history had no effect on the risk for neonatal hyperbilirubinemia. Neonatal hyperbilirubinemia was related with low E_3_ levels. The ratios of AFP/E_3_ and hCG/E_3_ were the most helpful to predict the neonatal hyperbilirubinemia.

**Conclusions:**

According to our results, low E_3_ levels in the triple test result can be helpful to predict the development of the neonatal hyperbilirubinemia. However, this is a bit expensive and many developing countries may not afford it.

## INTRODUCTION

Neonatal jaundice, also called as neonatal significant or severe hyperbilirubinemia, is the most common condition that requires hospital admission and outpatient follow-up after discharge in neonates. Neonatal physiologic jaundice can be a result of increased bilirubin production secondary to accelerated destruction of erythrocytes or decreased excretory capacity secondary to low levels of ligandin which is a binding protein for bilirubin in hepatocytes and low activity of bilirubin-conjugating enzyme uridine diphosphoglucuronyltransferase (UDPGT).

Although the level for definition varies according to gestational age, the values of more than 14 mg/dL in preterm infants and 17 mg/dL in term infants are accepted as neonatal significant hyperbilirubinemia. Many researchers have tried to find a predictor for this condition over the years. We hypothesized that abnormal function of fetal liver could help us the prediction of neonatal severe hyperbilirubinemia. As mentioned before, one of the pathophysiological mechanisms underlying neonatal hyperbilirubinemia may be related with abnormal functions in liver. For that reason, estriol (E_3_) and alpha-fetoprotein (AFP), second trimester serum markers, could be used as helpful markers. Because estriol constitutes 60-70% of the total estrogens during pregnancy if the fetal adrenal and liver are functional and AFP is secreted during pregnancy from the fetal liver in a very high amount.^1^ We aimed to define if there is any relationship between second trimester serum markers and neonatal severe hyperbilirubinemia to protect the neonates from its neurological damage.

## METHODS

This study is a retrospective case-control study. Total 1372 pregnant women were enrolled who had done triple test between April 2014 and April 2015 and then given birth at Kanuni Sultan Suleyman Training and Research Hospital. Healthy neonates with gestation ≥34 weeks were included. Multiple pregnancies, newborns with major congenital malformations or neural tube defect, patients with Rh incompatibility were excluded. All demographic and clinical characteristics including age, weight, obstetric history, presence of any comorbid conditions, in vitro fertilization pregnancy, smoking, gestational week during triple test and birth, birth weight, type of delivery, gender of baby, serum levels of E_3_, AFP and total bilirubin concentrations were obtained from written or electronic records. Gestational ages were estimated by ultrasonographic dating of the pregnancies. All maternal serum markers had been studied with solid-phase competitive enzyme immunoassay method. All results were converted into multiples of the median (MoM) for each of the analytes.

Our primary outcome was neonatal significant hyperbilirubinemia indicated by a bilirubin concentration of ≥17 mg/dL. Our study was designed retrospectively and conducted according to the Helsinki Declaration. There was not ethical approval because we collected data of the patients from the records and we did not documented any personal information. Also in our hospital, informed consent is taken from every patient about that medical information may be used in scientific publications.

### Statistical Analysis

Statistical analysis was performed with the Statistical Package for the Social Sciences (SPSS Inc; Chicago, IL, USA) statistics 22.0 version for Windows. Difference in mean values and characteristics between groups were analyzed with independent samples t test and chi-square test. Means were presented with standard deviation (SD). p<.05 was considered statistically significant. Thresholds for the association of abnormal maternal serum triple analytes with neonatal significant hyperbilirubinemia for this study were determined by initially using receiver operating characteristics (ROC) curves to ascertain the optimal cut-off for each analyte. When a significant cut-off value was observed, the sensitivity, specifity, positive likelihood ratio values were presented. While evaluating the area under the curve, a 5% type-I error level was used to accept a statistically significant predictive value of the test variables.

## RESULTS

Total 1372 women who had done triple test between April 2014 and 2015 and given birth at Kanuni Sultan Suleyman Training and Research Hospital were included. The mean age of our study population was 27.9±5.6, whereas the median age was 28. According to median age, the majority of women in the group aged 28-51. The mean gestational week was 17.3±1.0 during triple test, 38.3±2.7 during birth (Table-I). Most patients were multiparous giving birth by vaginal route and had no Rh incompatibility. A total of 59 patients had babies with neonatal hyperbilirubinemia after exclusion of Rh incompatibility as an important reason for neonatal hyperbilirubinemia (Table-II).

**Table-I:** Demographic and clinical characteristics of patients.

*Characteristics*	*Mean ± SD*
Age	27.9±5.6
Weight	65.7±12.8
Gravide	2.6±1.4
Parite	1.2±1.0
Gestational week on triple test	17.3±1.0
E_3_	1.00±0.40
hCG	1.17±0.72
AFP	0.99±0.55
Gestational week on birth	38.3±2.7
Birth weight	3168.9±629.9

E_3_, estriol; hCG, human chorionic gonadotropin; AFP, alpha-fetoprotein.

**Table-II:** Distribution of demographic and clinical characteristics.

*Characteristics*	*No. (%)*
***Age***		
≤28	676 (49.3)
>28	696 (50.7)
***Parite***	
0	346 (25.2)
≥1	1026 (74.8)
***Smoking***	
Absent	1223 (89.1)
Present	149 (10.9)
***Chronic health problems (DM, etc)***	
Absent	1351 (98.5)
Present	21 (1.5)
***In vitro fertilization pregnancy***	
Absent	1360 (99.1)
Present	12 (0.9)
***Poor obstetric history***	
Absent Present	1363 (99.3) 9 (0.7)
***Route of labor***	

Vaginal birth	788 (57.4)
Cesarean section	584 (42.6)
***Adverse obstetric result (preeclampsia, IUGR, GDM,etc)***	
Absent	1259 (91.8)
Present	113 (8.2)
***Rh incompatibility***	
Absent	1362 (99.3)
Present	10 (0.7)
***Neonatal hyperbilirubinemia***	
Absent	1313 (95.7)
Present	59 (4.3)

DM, diabetes mellitus; IUGR, intrauterine growth restriction; GDM, gestational diabetes mellitus.

We detected that the presence of in vitro pregnancy, maternal health problems or poor obstetric history had no effect on the risk for neonatal hyperbilirubinemia. There are differences between patients with or without neonatal hyperbilirubinemia based on clinical and demographic characteristics although there is not any statistical significance (Table-III).

**Table-III:** Comparison of characteristics of the patients according to presence of neonatal hyperbilirubinemia.

*Characteristics*	*Neonatal hyperbilirubinemia*		

	*Absent*	*Present*	*p*
***Age***			
≤28	641 (48.8)	35 (59.3)	NS
>28	672 (51.2)	24 (40.7)	
***Parite***			
0	332 (25.3)	14 (23.7)	NS
≥1	981 (74.7)	45 (76.3)	
***Smoking***			
Absent	1169 (89)	54 (91.5)	NS
Present	144 (11)	5 (8.5)	
***Chronic health problems (DM, etc)***			
Absent	1292 (98.4)	59 (100)	NS
Present	21 (1.6)	0
***In vitro fertilization pregnancy***			
Absent	1301 (99.1)	59 (100)	NS
Present	12 (0.9)	0	
***Poor obstetric history***			
Absent	1304 (99.3)	59 (100)	NS
Present	9 (0.7)	0
***Route of labor***			
Vaginal birth	752 (57.3)	36 (61)	NS
Cesarean section	561 (42.7)	23 (39)	
***Adverse pregnancy outcomes***			
Absent	1207 (91.9)	52 (88.1)	NS
Present	106 (8.1)	7 (11.9)	

DM, diabetes mellitus.

In our study, neonatal hyperbilirubinemia was related with low E_3_ levels. If the MoM values were compared with each other, the ratios of AFP/E_3_ and hCG/E_3_ were the most helpful to predict the neonatal hyperbilirubinemia (Table-IV).

**Table-IV:** Relationship between neonatal hyperbilirubinemia and MoM values.

	*Cut-off value*	*Area under curve*	*Sensitivity (%)*	*Specifity (%)*	*Positive likelihood ratio*
E_3_	0.435	0.45	98.3	2.5	1.01
	0.865		52.5	40.6	0.88
hCG	1.045	0.51	52.5	50.1	1.05
	1.115		50.8	55.2	1.13
AFP	0.845	0.48	54.2	44.3	0.97
	0.875		50.8	47.6	0.97
AFP/E_3_	0.95	0.53	59.3	50.2	1.20
	0.98		55.9	52.5	1.18
AFP/hCG	0.87	0.51	52.5	48.2	1.01
	0.94		50.8	53.8	1.10
hCG/E_3_	1.10	0.53	59.3	50.6	1.20
	1.11		57.6	51.6	1.19

E_3_, estriol; hCG, human chorionic gonadotropin; AFP, alpha-fetoprotein.

**Table-V:** Results of logistic regression analysis.

	*RR (95% CI)*	*p*
AFP	1.57 (1.17-2.09)	.002
hCG	1.09 (0.87-1.38)	NS
E_3_	0.74 (0.44-1.23)	NS
AFP/E_3_	1.38 (1.17-1.64)	<0.001
AFP/hCG	0.99 (0.90-1.08)	NS
hCG/E_3_	1.21 (1.05-1.40)	0.009

E_3_, estriol; hCG, human chorionic gonadotropin; AFP, alpha-fetoprotein; RR, risk ratio.

## DISCUSSION

Neonatal jaundice is the most common condition that requires medical attention in neonates. Neonatal physiologic jaundice can be a result of increased bilirubin production secondary to accelerated destruction of erythrocytes or decreased excretory capacity secondary to low levels of ligandin which is a binding protein for bilirubin in hepatocytes and low activity of bilirubin-conjugating enzyme uridine diphosphoglucuronyltransferase (UDPGT). Neonatal significant jaundice is defined as leveled of at 14 mg/dL at 4 days in preterm infants and 17 mg/dL in term infants requiring phototherapy or exchange transfusion to lower the total serum bilirubin concentration.^2^ Many factors including race, geography, genetics, nutrition, maternal factors such as drug usage, presence of diabetes mellitus, birth weight, gestational age and congenital infections can increase the incidence of pathologic neonatal jaundice.

Pathologic neonatal jaundice is one of the leading cause for hospital readmissions during the first week of postnatal life especially in low-income countries.^3^ This condition is important because increased bilirubin concentrations may result in deaths or various neurological impairments such as intellectual deficits, epilepsy, sensorineural hearing loss.^4^ The cause for these neurological deficits is accumulation of bilirubin in brain, termed as “kernicterus”. Early detection and management of kernicterus prevents these consequences. The best approach to prevent significant hyperbilirubinemia and its consequences is bilirubin screening before hospital discharge.^5^ But neonates with uncomplicated delivery mostly are discharged 48 hours after birth, so the diagnosis of significant hyperbilirubinemia may be delayed. Because of that, outpatient follow-up is critical for detection and management of kernicterus.

Many studies have been done to define an early marker of neonatal jaundice for the early detection of neonates at high risk of severe hyperbilirubinemia to prevent long-term poor results.^6^ Chou et al found a strong correlation between cord blood hydrogen peroxide levels and bilirubin concentrations.^7^ Alkaline phosphatase level has been thought as a significant predictor for the severe hyperbilirubinemia and bilirubin-induced neurological damage.^8^ Bilirubin/albumin ratio was thought as good a indicator of risk for bilirubin neurotoxicity.^9^

Triple test is applied between 14 and 21 gestational weeks and determine the risk for fetal chromosomal abnormalities combining maternal serum levels of AFP, E_3_, human chorionic gonadotropin (hCG) and maternal age although it is not diagnostic.^10^ There is also another benefit of triple test that many adverse pregnancy outcomes such as preeclampsia may be understood with these serum markers. The relationship between neonatal severe hyperbilirubinemia and serum levels of second trimester markers was investigated in our study. Because we thought that the neonatal hyperbilirubinemia which is an abnormality of the liver function could be understood with the level of serum E_3_, AFP, hCG and the ratio of these hormones during second trimester.

Our results showed that neonatal significant hyperbilirubinemia is associated with the low levels of E_3_ with a high sensitivity and low specificity. The absence of statistically significant relationship could be explained as many factors influence the levels of second trimester serum markers and the origin of E_3_ during pregnancy is primarily fetal adrenal rather than fetal liver.

According to our results, low E_3_ levels in the triple test result can be helpful to predict the development of the neonatal hyperbilirubinemia. Being aware the importance of early detection and management of neonatal hyperbilirubinemia, further studies should be needed to find predictors for that condition during pregnancy.

### Authors’ contributions

**HGC** study conception, protocol development, editing the manuscript and preparing the first draft of the article.

**EC** study conception, protocol development and data extraction.

**GY and MC** study design, revised and edited the manuscript.

All authors read and approved the final manuscript.
